# Patient, carer and health service outcomes of nurse-led early discharge after breast cancer surgery: a randomised controlled trial

**DOI:** 10.1038/sj.bjc.6601998

**Published:** 2004-07-06

**Authors:** M Wells, A Harrow, P Donnan, P Davey, S Devereux, G Little, E McKenna, R Wood, R Chen, A Thompson

**Affiliations:** 1 School of Nursing and Midwifery, University of Dundee, Dundee DD1 4HJ, UK; 2 Tayside Centre for General Practice, University of Dundee, Ninewells Hospital and Medical School, Dundee DD1 9SY, UK; 3 Medicines Monitoring Unit (MEMO), University of Dundee, Ninewells Hospital and Medical School, Dundee DD1 9SY, UK; 4 Tayside Lead Cancer Team, Roxburghe House, Royal Victoria Hospital, Dundee DD2 1SP, UK; 5 Acute Services Division, NHS Tayside, Ninewells Hospital and Medical School, Dundee DD1 9SY, UK; 6 Department of Epidemiology and Public Health, UCL, London WCIE 6BT, UK; 7 Department of Surgery and Molecular Oncology, University of Dundee, Ninewells Hospital and Medical School, Dundee DD1 9SY, UK

**Keywords:** breast cancer, axillary clearance, nurse led, early discharge

## Abstract

Patients with breast cancer who require axillary clearance traditionally remain in hospital until their wound drains are removed. Early discharge has been shown to improve clinical outcomes, but there has been little assessment of the psychosocial and financial impact of early discharge on patients, carers and the health service. This study aimed to evaluate the effectiveness of a nurse-led model of early discharge from hospital. Main outcome measures were quality of life and carer burden. Secondary outcomes included patient satisfaction, arm morbidity, impact on community nurses, health service costs, surgical cancellations and in-patient nursing dependency. A total of 108 patients undergoing axillary clearance with mastectomy or wide local excision for breast cancer were randomised to nurse-led early discharge or conventional stay. Nurse-led early discharge had no adverse effects on quality of life or patient satisfaction, had little effect on carer burden, improved communication between primary and secondary care, reduced cancellations and was safely implemented in a mixed rural/urban setting. In total, 40% of eligible patients agreed to take part. Nonparticipants were significantly older, more likely to live alone and had lower emotional well being before surgery. This study provides further evidence of the benefits of early discharge from hospital following axillary clearance for breast cancer. However, if given the choice, most patients prefer to stay in hospital until their wound drains are removed.

It is standard practice for patients undergoing axillary clearance for breast cancer to remain in hospital until their wound drains are removed. Randomised controlled trials carried out in Holland (Bonnema *et al*, 1998a) and England (Bundred *et al*, 1998; Horgan *et al*, 2000) have suggested that early discharge following axillary clearance has no adverse effect on physical or psychological outcome, and is acceptable to patients. There are indications that early discharge (with drains *in situ*) may enhance shoulder movement (Bundred *et al*, 1998), reduce wound pain (Bundred *et al*, 1998) and improve family support (Bonnema *et al*, 1998a), and it has been assumed that financial benefits to the Health Service will ensue.

Advocates of early discharge policies may be satisfied by these findings. However, five common fallacies exist in estimating the economic gains of early hospital discharge (Jonsson and Lindgren, 1990):

1. There are no adverse effects on the patient.
2. There is no burden for caregivers.
3. There are no costs imposed on primary care.
4. The hospital budget saves an amount equivalent to the average cost per day multiplied by the number of days saved.
5. Hospital waiting lists can be reduced correspondingly.

None of the existing studies has fully considered the ‘hidden’ psychosocial and financial costs to informal carers, for whom changes in the delivery of cancer services impose an increasing care burden (Fallowfield, 1998). There are indications that early discharge may simply transfer the burden to primary care (Bonnema *et al*, 1998b), although the views of staff working in the community have not been elicited. Studies have so far used limited questions to establish patient satisfaction with care, and none have employed breast cancer-specific tools to investigate the impact of hospitalisation or early discharge on quality of life. Moreover, almost two-thirds of the potential population of patients may be excluded on the grounds of geographical or social ineligibility (Bundred *et al*, 1998), thus making it difficult to estimate how many women facing axillary clearance surgery would, in reality, opt for early discharge if it was offered.

To test the hypothesis that nurse-led early discharge would not adversely affect quality of life or carer burden at 2 weeks after surgery, this study evaluated the impact of nurse-led early discharge following axillary clearance on patients, carers and the health service. The evaluation addressed key psychosocial and economic outcomes of a new model of care, implemented across the primary–secondary care interface.

## MATERIALS AND METHODS

**Figure 1:**
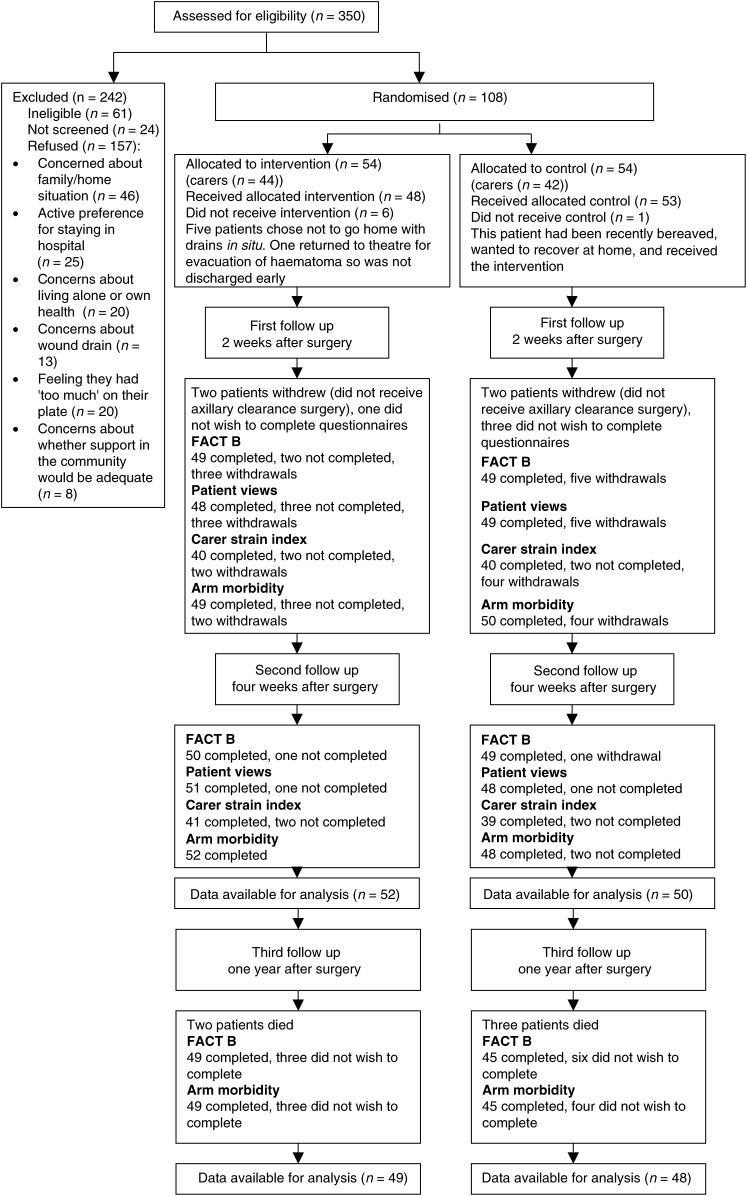
Flow chart of participating patients.

**Figure 2:**
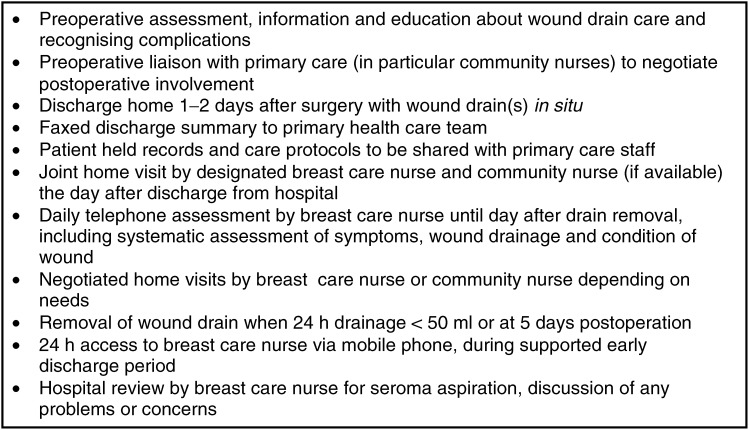
Essential components of the nurse-led model of care.

Female patients with breast cancer requiring level 1, 2 and 3 axillary clearance surgery (Thompson, 1999), diagnosed at a 910-bedded teaching hospital, were approached, provided that they were of good performance status (WHO⩽2), had no unstable medical conditions and had a telephone at home. Following screening for entry (Figure 1

), eligible patients were visited at home by the Research Assistant (AH) between diagnosis and surgery. A central telephone service provided by the Scottish Cancer Therapy Network Trials Office randomised consenting patients using a block randomisation technique. Owing to the nature of the intervention, it was not possible to blind participants, researchers or staff involved. Patients were stratified according to breast operation (mastectomy or wide local excision) to receive either:

- Nurse-led early discharge within 36 h of surgery, with wound drains still *in situ* (Figure 2

) *or*
- Conventional hospital stay following surgery until wound drains were removed (approximately 6 days)

All patients were asked to identify a main carer, who was also invited to consent to participate in the study.

The Tayside Committee for Research Ethics approved the protocol. Regular meetings were held with primary care and community nursing representatives, before and during the trial, to enhance collaboration across the primary–secondary care interface.

### Outcome measures

Complete data were collected at baseline, 2 weeks and 4 weeks. Data on quality of life and arm morbidity were also collected 1 year after surgery, by which time any adjuvant treatment would have been complete. Primary outcomes were quality of life, as measured by the Functional Assessment of Cancer Therapy-Breast (FACT-B) (Brady *et al*, 1997) and EQ-5D (Euroqol group, 1990) questionnaires, and carer burden. In order to acknowledge the contribution carers make to the patient's ‘care package’ (Thomas and Morris, 2002), carers were asked about their main concerns, and about the tasks and associated burden of caring during the first 4 weeks after surgery. The impact of caring was measured by the Carer Strain Index (Robinson, 1983). Patients’ views of care were assessed using a patient satisfaction questionnaire incorporating four dimensions: organisation of care, information and advice, personal experience of care and satisfaction with care (Moore *et al*, 2002). This questionnaire has face validity and, although tested on a different patient group, was primarily designed to assess satisfaction with the experience of a *model of care* (follow-up) provided after discharge from hospital, rather than a series of cancer site-specific concerns.

Wound healing and arm morbidity were measured at 2 and 4 weeks post surgery, and upper limb swelling and arm morbidity measured again at 1 year. Data on surgical cancellations and nursing dependency were collected before and during the trial, from the intervention ward (breast and general surgery) and a control ward (general surgery only). Questionnaires were sent to community nurses of all patients in the study and in depth interviews were carried out with the hospital-based breast care nurses to ascertain their views on early discharge or conventional stay. Demographic and quality of life data were collected from all patients attending the preoperative assessment clinic to enable comparison between participants and nonparticipants. Reasons for refusing entry into the trial were also elicited.

We performed a pragmatic cost analysis, in which key costs and consequences were identified for all stakeholders (NHS, carers and patients), measured and presented in their natural units. The perspective for this analysis was societal. In addition, we performed an analysis from the perspective of the NHS, with bed days saved and cancelled operations avoided as the measures of effect. Resource costs (mainly staff time) were obtained from NHS Tayside.

### Statistical methods

Characteristics of patients in each arm were compared descriptively according to CONSORT guidelines (Moher *et al*, 2001). Deprivation categories were calculated from postcodes using Carstairs scores obtained from the 1991 census (McLoone, 1991). Analysis was performed on an intention to treat basis.

Quality of life scores were calculated according to the FACIT (Cella, 1997) and EUROQOL (Euroqol group, 2000) scoring manuals. The patient satisfaction questionnaire was analysed using a similar method, calculating subscores for each domain of satisfaction (Moore *et al*, 2002). Differences in arm morbidity, quality of life, carer burden and patient satisfaction were tested using *χ*^2^ (for categorical variables) and *t*-tests, or Mann–Whitney *U*-tests for nonparametric data. Likelihood ratio tests were used where the numbers in contingency tables were small. Repeated measures analysis of FACT scores and carer strain were carried out along with Mauchly's test for sphericity. Arm volume data were analysed using regression analysis of the change in volume of the affected arm during the year after surgery, adjusting for baseline volume, change in volume of the unaffected arm and whether or not the patient had undergone adjuvant therapy. Cancellations and dependency scores were analysed using regression analyses including time trends and lagged variables. Cancellation counts were modelled as Poisson distributions, while dependency scores were normally distributed. Both measures were compared before and after the intervention and with the control ward. These analyses were carried out in SPSS or S-plus.

Our original hypothesis was that nurse-led early discharge would not adversely affect quality of life or carer burden at 2 weeks. We had calculated that a total sample size of 224 patients would be necessary to detect a 8% or more (9.2 points) difference in quality of life between the two arms, assuming a two-sided 5% significance level, a power of 90% and a standard deviation of 20.9 (Brady *et al*, 1997). However, we found that 59% of women in our population were unwilling to accept randomisation and thus 108 women were recruited, providing 80% power to detect differences of 10% or more (11.3 points) in FACT-B scores between groups. Recruitment commenced in April 2000 and ended in August 2002. Withdrawals from the study were minimal and data compliance excellent (Figure 1).

## RESULTS

### Baseline characteristics

Baseline characteristics of patients (*n*=108) and carers (*n*=86) were similar for both groups (Table 1

**Table 1:** Baseline characteristics

**Characteristic**	**Early discharge (*n*=54)**	**Conventional stay (*n*=54)**
	**Mean (s.d.)**	**Mean (s.d.)**
		
Age	54.9 (12.23)	57.3 (8.66)
Number of years full-time education after 13 years	4.3 (2.71)	3.8 (2.68)
		
	**No. (%)**	**No. (%)**
*Type of surgery*		
Wide local excision	29 (53.7)	29 (53.7)
Mastectomy	25 (46.3)	25 (46.3)
*Deprivation category*		
Most affluent-1	7 (13)	3 (5.9)
2	15 (27.8)	11(21.6)
3	15 (27.8)	17 (33.3)
4	6 (11.1)	10 (19.6)
5	4 (7.4)	3 (5.9)
6	7 (13)	7 (13.7)
*Comorbid disease*		
Combined (all)	35 (64.8)	32 (59.3)
Cardiovascular risk factors	14 (25.9)	9 (16.7)
Chest problems	9 (16.7)	5 (9.3)
Arthritis, back pain	8 (14.8)	7 (13)
Endocrine disorders	7 (13)	7 (13)
Noninsulin diabetic	2 (3.7)	1 (1.9)
Skin disorders	1 (1.9)	3 (5.6)
Gastrointestinal disease	3 (5.6)	4 (7.4)
Other	13 (24.1)	17 (31.5)
*World health organisation performance status*		
Normal activity without restriction	50 (92.6)	51 (94.4)
Strenuous activity restricted, can do light work	4 (7.4)	3 (5.6)
*Marital status*		
Single (never married)	3 (5.6)	1 (1.9)
Married	40 (74.1)	38 (70.4)
Widowed	5 (9.3)	5 (9.3)
Divorced/separated	6 (11.2)	10 (18.5)
Living alone	6 (11.1)	7 (13)
Living with others	48 (88.9)	47 (87)
Carer available – all day/night	47 (87)	44 (81.5)
Carer available – as required	7 (13)	10 (18.5)
*Carer self-rating of general health*	*n*=44	*n*=42
Poor	1 (2.3)	2 (2.4)
Fair	3 (6.8)	5 (12.2)
Good	25 (56.8)	25 (61)
Excellent	15 (34.1)	10 (24.4)
*Carer Comorbidity*		
All	16 (36.4)	17 (39.5)
		
	**Mean (s.d.)**	**Mean (s.d.)**
Age of nominated carer	60.33 (18.83)	59.11 (18.36)
Distance from hospital (miles)	14.2 (11.27)	16.5 (13.88)
*FACT subscale scores*		
Physical well being	25.4 (2.95)	24.8 (3.17)
Social and functional well being	24.4 (4.35)	24.9 (2.92)
Emotional well being	16.9 (4.14)	16.07 (5.32)
Functional well being	22.02 (5.34)	20.8 (5.85)
*FACT G total score*	88.7 (12.47)	86.6 (12.47)
Additional concerns (breast score)	26.6 (4.9)	26.1 (4.98)
FACT B total score^a^	115.3 (15.31)	113.4 (15.1)
EUROQOL		
Health state^b^	78.3 (16.42)	78.1 (18.59)
EQ-5D index^c^	0.81 (0.21)	0.79 (0.20)
	Median 0.85	Median 0.85
		
	**No. (%)**	**No. (%)**
*EUROQOL*		
Mobility – no problems	48 (90.6)	43 (81.1)
Self-care – no problems	52 (98.1)	48 (90.6)
Usual activities – no problems	43 (81.1)	43 (81.1)
No pain or discomfort	37 (71.1)	35 (66)
No anxiety or depression	20 (37.7)	20 (37.7)
*Movement of affected arm*		
Flexion – full	52 (96%)	54 (100%)
Extension – full	53 (98%)	53 (98%)
Abduction – full	51 (94%)	53 (98%)
Lateral rotation –full	40 (74%)	42 (78%)
Medial rotation – full	49 (91%)	46 (85%)

a FACT B=FACT G+additional concerns (breast). Higher scores represent better quality of life.

b Measured using 100 mm visual analogue scale. Higher scores=better quality of life.

c A weighted health index, where full health has a value of 1 and death a value of 0.

). Nonparticipants were older (61.4 years (95% CI 59.3–63.5) *vs* 56.2 years (95% CI 54–58) *P*<0.0001) and more likely to live alone (23 (21%) *vs* 13 (12%) *P*=0.071). They also had lower scores for emotional well being as measured by FACT-B (14.4 (95% CI 13.3–15.5) *vs* 16.7 (15.8–17.6) *P*=0.001) and poorer quality of life (EQ5D mean 0.76 (s.d. 0.195) *vs* 0.80 (s.d. 0.204) *P*=0.036). There were no significant differences between participants and nonparticipants in the type of surgery, distance from hospital or deprivation category.

Reasons patients gave for declining entry into the study included;

- concern that it would put too much responsibility on the family
- worries about small children, dogs, living alone and other home circumstances
- fears that the community support might not be adequate
- having ‘too much on their plate’ already
- an active preference for staying in hospital to recover.

### Comparison between nurse-led early discharge and conventional stay

Mean hospital stay was 3.96 days in the early discharge group compared with 6.22 days in the control group (95% CI difference −2.74 to −1.78 days). There were two readmissions to hospital in each group. Patients who were discharged early received significantly more home care (Table 2

**Table 2:** Time and number of visits/contacts with district nurses (DN), general practitioners (GP) and breast care nurses (BCN) during the first 4 weeks after surgery

	**Early discharge**		**Conventional stay**		**Mann**
	**Mean (s.d.)**	**Median**	**Mean (s.d.)**	**Median**	**Whitney**
					***P*-value**
No. of home visits by DN	3.1 (3.9)	1.0	0.6 (1.8)	0.0	<0.001
No. of home visits BCN	1.7 (0.9)	2.0	0.1 (0.7)	0.0	<0.001
Mileage for home visits (BCN)	39.2 (34.4)	34.0	1.1 (5.6)	0.0	<0.001
Time (mins) home visits (BCN)	132.1 (102)	120	0.0	0.0	—
Time (mins) telephone assessments (BCN)	17 (13.9)	15	1.98 (4.3)	0.0	<0.001
No. of home visits by GP	0.5 (0.8)	0.0	0.3 (0.7)	0.0	0.165
No. of visits to GP	0.7 (0.8)	1.0	0.9 (0.8)	1.0	0.079
Cost additional prescriptions	4.1 (8.1)	0.0	4.0 (6.5)	0.0	0.948

).

**Figure 3:**
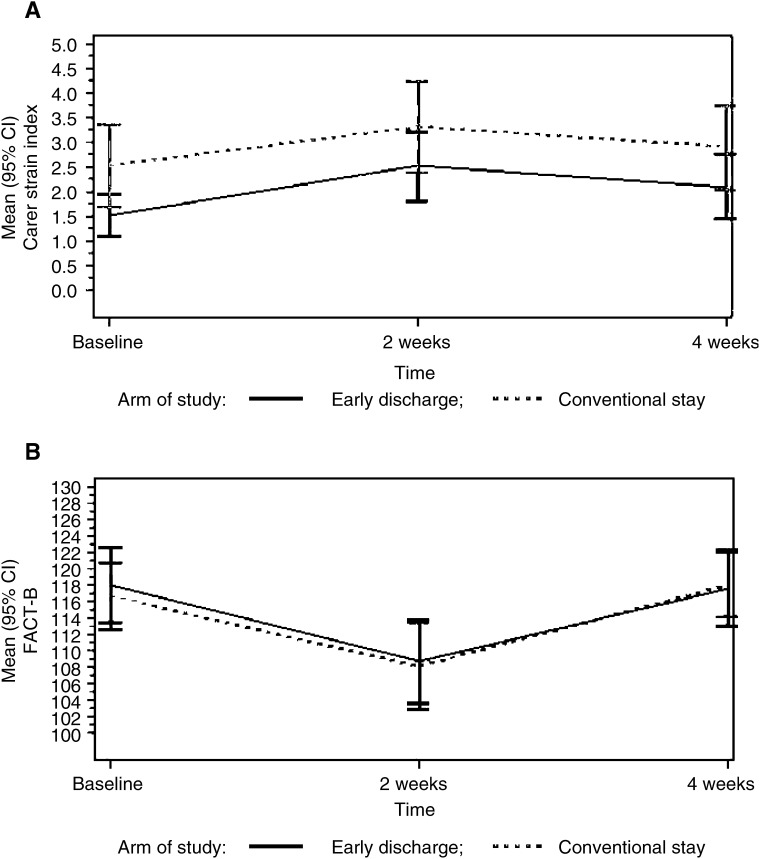
Change in mean levels of (**A**) carer strain scores and (**B**) FACT B quality of life scores over 4 weeks following axillary clearance surgery. No significant difference between arms of the study over time.

No differences were found between groups, at any time points, in repeated measures analysis of quality of life (FACT-B) or carer strain scores (Figure 3

) although initial carer strain scores (measured after the outcome of randomisation was known) were higher in the conventional stay group. At 1 year, there was no change in quality of life from 4 week levels. There were no significant differences in EUROQOL domains or EQ-5D scores at 2 weeks, 4 weeks or 1 year. Equal numbers of patients in both randomised groups received chemotherapy, radiotherapy or hormone therapy during the year after surgery.

Time taken off work by carers was similar in both groups (mean hours 24.07 (95% CI 8.63–39.51) for early discharge *vs* mean hours 29.11 (95% CI 6.76–51.46) for conventional stay). More carers in the early discharge group reported having to assist with wound care (56 *vs* 17% in the conventional stay group; *P*<0.0001), but there were no other significant differences in the extent to which carers had to help with activities of daily living. In the early discharge group, 90% of carers said they would choose the same care again compared with 78% in the control group (*P*<0.0001) and 65% reported that their lives had been disrupted as a result of caring, compared with 74% of carers in the control group. When asked about their main concerns during the postoperative period, a greater proportion of carers in the conventional stay group (69 *vs* 40%) provided comments at both 2 weeks and 4 weeks after surgery. Carers were particularly worried that their wives were doing too much around the house, and were concerned about wound healing, pain and waiting for pathology results. Several also commented on the lack of contact with health professionals and apparent lack of communication between the hospital and GP. The concerns of carers in the early discharge arm mainly centred around wound care, dressings and drain safety, travelling back to hospital for drainage of seromas, the worry of waiting for results and the emotional support they needed to give their wives. At 4 weeks after surgery, carers in both groups were most concerned about their wives emotional well being, but several of those in the conventional stay group also made comments about the lack of professional support and worries about wound healing.

Axillary wound drains remained *in situ* longer in those who were discharged early (mean 5.14 days (95% CI 4.77–5.5) *vs* 4.59 days (95% CI 4.18–5.01). Two patients in the early discharge group and one in the conventional stay group returned to theatre for evacuation of a haematoma before they were discharged. There were more seroma aspirations in the conventional stay group (median 1.5 *vs* 1). At 2 weeks postoperatively, conventional stay patients were significantly more likely to have a wound infection and be on antibiotics (nine patients (18.4%) *vs* two patients (4%) *P*=0.019), but there were no significant differences between the groups at 4 weeks. The conventional stay group had worse scores for pain and numbness in the breast, arm or axilla, but these differences were not statistically significant at any time point. Analgesia use overall was similar in both groups. More patients in the early discharge group were able to bend their affected arm behind their back (medial rotation: as if doing up a bra) at 2 weeks postoperatively (46, 94% *vs* 41, 82%; *P*=0.096). This nonsignificant difference remained at 1 year (42, 86% *vs* 33, 69%; *P*=0.131).

Patients in the early discharge group had a slightly greater increase in arm volume than patients in the conventional stay group (248.1 cm^3^ (95% CI 178.5–317.7) *vs* 157.6 cm^3^ (95% CI 77.5–237.6)). As a percentage increase, this difference was 9.9% (95% CI 6.8–12.9) in the early discharge group and 7% (95% CI 2.7–11.4) in the conventional stay group. It was neither statistically (*P*=0.053) nor clinically significant.

Patients’ scores for each of the four domains of care were almost equivalent between groups and, on a scale from 0 to 10, mean overall rating of care was 8.79 (95%CI 8.44–9.15) in the early discharge group and 8.36 (95% CI 7.84–8.88) in the control group at 2 weeks.

More patients in the early discharge group would have opted again for the care they received (88 *vs* 69% of the control group; *P*<0.0001). Patients in the early discharge group valued the opportunity to be at home with their families, and those in conventional stay particularly commented on the support they received from other patients in the ward. Both groups were positive about the care they had received, although the early discharge patients were concerned about the withdrawal of home support after the removal of the drain, and disliked having to return to the hospital for seroma aspirations. Conventional stay patients expressed more frustration with the restrictions of being in hospital.

### Impact on primary and secondary care

We received questionnaires from 64 out of 100 community nurses, of whom the majority (41) were caring for patients in the early discharge group. Community nurses of patients in the conventional stay group were much less likely to know which type of care their patient had received postoperatively (2 *vs* 65% in early discharge group; *P*<0.0001) or to have received information about the patient before surgery (4 *vs* 40%) or after surgery (13 *vs* 83%; *P*<0.0001). Only nine out of 23 (39%) community nurses of patients in the conventional stay group were aware that their patient had been discharged from hospital. In the early discharge group, 34 (83%) community nurses rated the ease of access to a specialist member of hospital staff as at least adequate and 25 (71%) said they would prefer nurse-led early discharge for patients in the future.

Regression analysis of cancelled operations revealed a reduction by 50% in the intervention ward (RR=0.50, 95% CI 0.41–0.61) compared with 34% in the control ward (RR=0.66, 95% CI 0.51–0.85) adjusted for a 1-month lag in counts. After additional adjustment for trend over time, the change only remained significant for the intervention ward (RR=0.65, 95% CI 0.46–0.91).

The overall mean nursing dependency score was greater in the control ward (mean difference *before* −0.25 (s.d. 0.039) and *after* intervention −0.401 (s.d. 0.055) *P*<0.001). In the regression analysis, there was a significant decrease in mean dependency scores in the intervention ward whereas there was a trend towards increased dependency scores in the control ward following the intervention.

All costs to the NHS of the nurse-led model of care were quantified, and the mean total cost was £183.83 per patient (Table 3

**Table 3:** Summary of main additional costs of nurse-led early discharge to the NHS

**Activity**	**Resource consumed**	**Cost per patient** (£)	**Comment**
Development of protocols	12 h F Grade & 8 h H Grade total time	5.06	Initial phase of study only
Preoperative education	45 min per patient	9.24	F Grade, home visit
Discharge preparation	75 min per patient	15.40	F Grade
Postoperative visits	Additional weekend shift cover by F, G and H grade nurses	85.06	Total cost of additional weekend cover by F, G and H grade nurses £4593.21
	Weekday, average of 66 min of F grade nurse per patient including travel	13.58	The average time for all postoperative visits including travel was 132 min per patient, but the time required for weekend visits has already been accounted for
Travel costs for home care	39.2 miles per patient	15.60	Postoperative home visit(s)
Telephone calls	Nurse time, 17 min per patient	3.49	
	Call charges	1.90	Includes local and national rates
Mobile phone hire	£8.23 per month of the study	3.66	
District nurse visits	87.6 min per patient	30.84	
			
Total NHS cost		183.83	

). Nurse-led early discharge saved 2.26 bed days per patient (£81.34 per bed day gained) and was associated with a reduction in cancellations of 2.9 per month over the 21 months of the study (£163.00 per cancellation avoided).

## DISCUSSION

Axillary clearance remains the standard surgical procedure for early breast cancer, despite the option of axillary node sampling (Steele *et al*, 1985) and the advent of sentinel node biopsy (McIntosh and Purushotham, 1998). Although it is possible to discharge patients early *without* a wound drain *in situ* (Purushotham *et al*, 2002), this has not become standard practice. This trial provides further evidence that early discharge for patients recovering from axillary clearance for breast cancer is safe (Bonnema *et al*, 1998a; Bundred *et al*, 1998; Horgan *et al*, 2000). In addition, this study provides novel data on patients, the applicability of early discharge to an unselected population of patients with breast cancer, and the benefits and burdens of early discharge or conventional stay for carers and primary or secondary health care services. It also demonstrates that there are no adverse long-term effects of early discharge, 1 year after surgery.

It is evident that early discharge increases the workload of community nurses and breast care nurses, and may increase patients’ expectations of what should be available in the postoperative period. The small and unpredictable number of patients receiving early discharge resulted in considerable organisational difficulties for the breast care nursing team, who were required to provide an irregular weekend service for relatively few patients on top of their existing workload. However, carers of patients being discharged early may feel less burdened in anticipation of surgery and our model of care also enhanced communication and teamwork between nurses in secondary and primary care.

We have shown that introducing early discharge, even for 20% of the possible patient population, was associated with a significant reduction in surgical cancellations on the intervention ward without increasing nursing dependency. This is the first direct demonstration of the productivity of saved resources within a randomised trial. The value of saved healthcare resources lies in their alternative uses (Jonsson and Lindgren, 1990) and the evidence from this study suggests that a nurse-led model of care is more cost-effective than a conventional service, even with the required investment in specialist nursing teams. Increasing the number of patients undergoing early discharge could increase the impact on cancellations, while at the same time producing economies of scale. For example, weekend home visits were covered through fixed shifts and the nurses could have accommodated additional visits at minimal marginal cost. While early discharge from hospital was just one component of our nurse-led model of care, this study provides information on the likely costs of early discharge in the context of a nurse-led service, a complex intervention with several interlocking components (Ukoummunne *et al*, 1999; Campbell *et al*, 2000; Medical Research Council, 2000).

Clearly, our results apply to the 40% who were prepared to be discharged home. A majority (60%) of patients declined the trial, these patients were older, more anxious/depressed and more likely to be living alone. In the context of a growing elderly population and a pressure to reduce hospital stay, these findings present a difficult dilemma for health care providers, and it remains to be seen whether our results will change the opinion of the majority of patients with breast cancer who would prefer to stay in hospital until surgical drains are removed. However, overall, nurse-led early discharge following axillary clearance for breast cancer is safe, carries an acceptable burden on carers and has significant overall benefits to patients and the breast service across the primary secondary care interface.

## References

[bib1] Bonnema J, van Wersch A, van Geel A, Pruyn J, Schmitz P, Paul M, Wiggers T (1998a) Medical and psychosocial effects of early discharge after surgery for breast cancer: randomised trial. BMJ 316: 1267–1271 9554895 10.1136/bmj.316.7140.1267 PMC28526

[bib2] Bonnema J, van Wersch A, van Geel A, Pruyn J, Schmitz P, Uyl-de Groot C, Wiggers T (1998b) Cost of care in a randomised trial of early hospital discharge after surgery for breast cancer. Eur J Cancer 34: 2015–2020 10070303 10.1016/s0959-8049(98)00258-5

[bib3] Brady M, Cella D, Mo F, Bonomi A, Tulsky D, Lloyd S, Deasy S, Cobleigh M, Shiomoto G (1997) Reliability and validity of the functional assessment of cancer therapy-breast quality-of-life instrument. J Clin Oncol 15: 974–986 9060536 10.1200/JCO.1997.15.3.974

[bib4] Bundred N, Maguire P, Reynolds J, Grimshaw J, Morris J, Thomson L, Barr L, Baildam A (1998) Randomised controlled trial of effects of early discharge after surgery for breast cancer. BMJ 317: 1275–1279 9804712 10.1136/bmj.317.7168.1275 PMC28705

[bib5] Campbell M, Fitzpatrick R, Haines A, Kinmonth A, Sandercock P, Spiegelhalter D, Tyrer P (2000) Framework for design and evaluation of complex interventions to improve health. BMJ 321: 694–696 10987780 10.1136/bmj.321.7262.694 PMC1118564

[bib6] Cella D (1997) FACIT Manual. Manual of the Functional Assessment of Chronic Illness Therapy (FACIT) Measurement System. Center on Outcomes, Research and Education (CORE) Evanston: Evanston Northwestern Healthcare and Northwestern University.

[bib7] Euroqol group (1990) Euroqol: a new facility for the measurement of health related quality of life. Health Policy 16: 199–208 10109801 10.1016/0168-8510(90)90421-9

[bib8] Euroqol group (2000) EQ-5D: a Measure of Health Related Quality of Life Developed by the EUROQOL Group. Rotterdam: Erasmus University

[bib9] Fallowfield L (1998) Early discharge after surgery for breast cancer. BMJ 317: 1264–1265 9804708 10.1136/bmj.317.7168.1264 PMC1114200

[bib10] Horgan K, Benson E, Robertson A (2000) Early discharge with drain *in situ* following axillary lymphadenectomy for breast cancer. The Breast 9: 90–92 14731706 10.1054/brst.2000.0142

[bib11] Jonsson B, Lindgren B (1990) Five common fallacies in estimating the economic gains of early discharge. Soc Sci Med 14: 27–33 10.1016/0160-7995(80)90005-2 7444483

[bib12] McIntosh S, Purushotham A (1998) Lymphatic mapping and sentinel node biopsy in breast cancer. Br J Surgery 85: 1347–1356 10.1046/j.1365-2168.1998.00934.x 9782012

[bib13] McLoone P (1991) Carstairs Codes for Scottish Postcode Sectors From the 1991 Census. Glasgow: Public Health Research Unit, University of Glasgow

[bib14] Medical Research Council (2000) A Framework for development and evaluation of RCT's for complex interventions to improve health. Medical Research Council

[bib15] Moher D, Schulz KF, Altman D, for the CONSORT Group (2001) The CONSORT Statement: revised recommendations for improving the quality of reports of parallel-group randomized trials. J Am Med Assoc 285: 1987–1991 10.1001/jama.285.15.1987 11308435

[bib16] Moore S, Corner J, Haviland J, Wells M, Salmon E, Normand C, Brada M, O’Brien M, Smith I (2002) Reconfiguring cancer follow-up care: a randomised trial comparing an alternative model of nurse led follow-up with conventional medical follow-up in the management of patients with lung cancer. BMJ 325: 1145 12433764 10.1136/bmj.325.7373.1145 PMC133453

[bib17] Purushotham A, McLatchie E, Young D, George W, Stallard S, Doughty J, Brown D, Farish C, Walker A, Millar K, Murray G (2002) Randomized clinical trial of no wound drains and early discharge in the treatment of women with breast cancer. Br J Surgery 89: 286–292 10.1046/j.0007-1323.2001.02031.x 11872051

[bib18] Robinson B (1983) Validation of a caregiver strain index. J Gerontol 38: 344–348 6841931 10.1093/geronj/38.3.344

[bib19] Steele R, Forrest A, Gibson T, Stewart H, Chetty U (1985) The efficacy of lower axillary sampling in obtaining lymph node status in breast cancer: a controlled randomized trial. Br J Surg 72: 368–369 3888336 10.1002/bjs.1800720512

[bib20] Thomas C, Morris S (2002) Informal carers in cancer contexts. Eur J Cancer 11: 178–182 10.1046/j.1365-2354.2002.00336.x 12296834

[bib21] Thompson A (1999) Axillary node clearance for breast cancer. J Roy Coll Surg Edin 44: 111–116 10230206

[bib22] Ukoummunne OC, Guilford MC, Chinn S, Sterne JAC, Burney PG, Donner A (1999) Methods in health service research: evaluation of health interventions at area and organisation level. BMJ 319: 376–379 10435968 10.1136/bmj.319.7206.376 PMC1126996

